# A Model for Estimating Dose-Rate Effects on Cell-Killing of Human Melanoma after Boron Neutron Capture Therapy

**DOI:** 10.3390/cells9051117

**Published:** 2020-04-30

**Authors:** Yusuke Matsuya, Hisanori Fukunaga, Motoko Omura, Hiroyuki Date

**Affiliations:** 1 Nuclear Science and Engineering Center, Research Group for Radiation Transport Analysis, Ibaraki 319-1195, Japan; 2 Faculty of Health Sciences, Hokkaido University, Hokkaiddo 060-0812, Japan; date@hs.hokudai.ac.jp; 3 Department of Radiation Oncology, Shonan Kamakura General Hospital, Kanagawa 247-8533, Japan; hfukunaga01@qub.ac.uk (H.F.); momuram@mac.com (M.O.)

**Keywords:** boron neutron capture therapy (BNCT), microdosimetry, dose-rate effects

## Abstract

Boron neutron capture therapy (BNCT) is a type of radiation therapy for eradicating tumor cells through a ^10^B(n,α)^7^Li reaction in the presence of ^10^B in cancer cells. When delivering a high absorbed dose to cancer cells using BNCT, both the timeline of ^10^B concentrations and the relative long dose-delivery time compared to photon therapy must be considered. Changes in radiosensitivity during such a long dose-delivery time can reduce the probability of tumor control; however, such changes have not yet been evaluated. Here, we propose an improved *integrated microdosimetric-kinetic model* that accounts for changes in microdosimetric quantities and dose rates depending on the ^10^B concentration and investigate the cell recovery (dose-rate effects) of melanoma during BNCT irradiation. The integrated microdosimetric–kinetic model used in this study considers both sub-lethal damage repair and changes in microdosimetric quantities during irradiation. The model, coupled with the Monte Carlo track structure simulation code of the Particle and Heavy Ion Transport code System, shows good agreement with in vitro experimental data for acute exposure to ^60^Co γ-rays, thermal neutrons, and BNCT with ^10^B concentrations of 10 ppm. This indicates that microdosimetric quantities are important parameters for predicting dose-response curves for cell survival under BNCT irradiations. Furthermore, the model estimation at the endpoint of the mean activation dose exhibits a reduced impact of cell recovery during BNCT irradiations with high linear energy transfer (LET) compared to ^60^Co γ-rays irradiation with low LET. Throughout this study, we discuss the advantages of BNCT for enhancing the killing of cancer cells with a reduced dose-rate dependency. If the neutron spectrum and the timelines for drug and dose delivery are provided, the present model will make it possible to predict radiosensitivity for more realistic dose-delivery schemes in BNCT irradiations.

## 1. Introduction

Radiation therapy is one of the treatment approaches for eradicating tumors in clinical practice [1]. Among several clinical modalities such as 6MV-linac X-ray, proton, carbon ion, and neutron capture therapies [2,3,4,5,6,7], boron neutron capture therapy (BNCT), in which ^10^B is administered to tumor cells [8], is one of the most effective approaches for treating malignant tumors. Due to the high linear energy transfer (LET) particles with a short range within approximately 10 μm (i.e., 1.47 MeV α particle and 0.84 MeV ^7^Li ion in 94% captures) that are emitted during the ^10^B(n,α)^7^Li reaction [9], the thermal neutron irradiation causes substantial damage to cells that take up the tumor-seeking ^10^B compounds, actualizing tumor-cell-selective killing. Boron neutron capture therapy has shown to have significant potential for treating cancers such as melanoma, brain tumors, and head and neck cancers. However, it has not been routinely applied in clinical practice because, for a long time, availability was limited to facilities with nuclear reactors. The advancement of BNCT requires neutron sources that can be installed in hospital environments. Further to the development of neutron accelerators, in recent experimental and clinical studies, accelerator-based BNCT systems have been installed in a small number of hospitals [10]. Therefore, BNCT for cancer treatment will become available at several medical institutes around the world that are equipped with accelerator-based BNCT modalities.

There are two major boron compounds available for BNCT, ^10^B-boronphenylalanine (BPA: C_9_H_12_BNO_4_) and ^10^B-sodium borocaptate (BSH: Na_2_B_12_H_11_SH) [11]. Although many other compounds have higher affinities to the tumors, they have not yet been used because of their toxicity and low tumor-to-normal-tissue ratios. In particular, the possibility of using BNCT on melanoma (and metastatic melanoma) using BPA has been experimentally and clinically reported [12,13]. When BPA with improved solubility is injected intravenously [14], it can be taken up by tumor cells through amino acid transporters on the cell membrane surface. Thus, BPA can enhance the selective killing of tumor cells; however, a precise understanding of the curative effects of BNCT is lacking due to the complexity of the treatment conditions, such as the timing of drug-delivery and the relatively long dose-delivery time in BNCT (e.g., 40 min or longer) compared to in photon therapy.

To evaluate the probability of tumor control after the administration of external radiation beams, the linear-quadratic (LQ) model [15,16,17] is widely used to extrapolate the experimental dose–response curve for cell survival data for each LET radiation. By contrast, the microdosimetric-kinetic (MK) model [18,19] enables the prediction of the LET-dependence of cell killing using microdosimetric quantities, such as dose-mean lineal energy *y_D_* in keV/μm [20], which has been tested by comparing with in vitro experimental data [21,22,23,24,25,26]. The microdosimetric quantities can be easily obtained from Monte Carlo simulations for radiation transport [21,27,28]. While cell recovery during dose delivery (dose-rate effects) with low-LET radiation at a constant dose-rate has been effectively evaluated in terms of sub-lethal damage repair (SLDR) [29,30,31], many available models so far (including the original MK model [19]) for predicting cell recovery are insufficient for BNCT. This is because those models do not consider both changes in the dose-rate and the microdosimetric quantities depending on ^10^B concentrations in tumor cells during the relatively long dose-delivery period [31,32]. Therefore, we are interested in developing a model that considers changes in ^10^B concentrations during dose delivery.

In this study, we propose a mathematical model for describing cell survival that calls into account both changes in microdosimetric quantities and dose rate. Our *integrated microdosimetric-kinetic (IMK) model* is unique in its incorporation of several biological factors [33,34,35,36] (i.e., dose-rate effects [33,34], intercellular communication [35,36] and cancer stem cells [36]). The IMK model enables us to describe the dose–response curve for cell survival modified by changes in radiation quality and dose rate during irradiation. In this paper, we present an example of radiosensitivity dynamics during BNCT irradiation, thereby contributing to enabling the radiosensitivity to be predicted for more realistic dose-delivery schemes in BNCT.

## 2. Materials and Methods

### 2.1. Calculation of Microdosimetric Quantities

To estimate the killing of melanoma cells after irradiation with BNCT, we performed Monte Carlo simulations and calculated the microdosimetric quantities of dose-mean lineal energy *y_D_* in keV/μm and saturation-corrected dose-mean lineal energy y* in keV/μm. The Monte Carlo simulation code of “Particle and Heavy Ion Transport code System (PHITS)” version 3.11 [28] adapting the electron gamma shower (EGS) mode [37] and event generator mode (e-mode = 2) [38] was used to calculate the *y_D_* and y* values. It should be noted that the y* value for photon beams is almost the same as the *y_D_* value, so we used the well-verified *y_D_* value of ^60^Co γ-rays reported previously (*y_D_* = 2.26 keV/μm) [34]. The cutoff energies of the neutrons and other radiation particles in PHITS were set to 0.1 eV and 1.0 keV, respectively.

The simulation geometry for an in vitro experiment with a petri dish for cell culture (i.e., 30 mm diameter × 15 mm height, plastic (^1^H:^12^C = 2:1) as component, 1.07 g/cm^3^ as density) containing culture medium (liquid water) with 2 mm thickness was considered in the PHITS code. Because of the difficulty in reproducing the same irradiation condition as the in vitro experimental condition [39], we used one of the thermal neutron beam spectra reported in the literature [40] and transported the neutrons. It should be noted that we also considered hydrogen captures in the dish and the contribution of the emitted photons to the microdosimetric quantities. The probability densities of lineal energy *y* and dose within a site with a 1.0 μm diameter were determined by sampling with a tally named “*t-sed*”, as reported previously [27,28]. We then calculated the *y_D_* and y* values using the following equations:

(1) $$y_{D}=\int yd\left(y\right)dy=\frac{\int y^{2}f\left(y\right)dy}{\int yf\left(y\right)dy},$$

(2) $$y*=\int \frac{1}{y}[1-\exp(y^{2}/{y_{0}}^{2})]d\left(y\right)dy,$$

where *y* is the lineal energy in keV/μm; *f(y)* and *d(y)* are the probability densities of lineal energy and dose, respectively; and *y*_0_ is a so-called saturation parameter to express the overkill effect [21,27]; the *y*_0_ value is obtained as 150 keV/μm in a previous report on the MK model [21,27].

### 2.2. Model Overview

#### 2.2.1. Improvement of the IMK Model to Consider Changes in ^10^B Concentrations

We modified the integrated microdosimetric-kinetic (IMK) model [33,34,35], which was based on DNA targeted effects, to incorporate the changes in the microdosimetric quantities of y* depending on ^10^B concentration dynamics after the intravenous injection of boron agents.

In the IMK model, the cell nucleus is sub-divided into multiple micro-order territories (domains) to incorporate microdosimetry [20]. The domains are generally defined as simple spheres with a 1.0 μm diameter [19,41], which corresponds to the PHITS simulation for sampling the lineal energy distribution. Radiation-induced DNA lesions that may be toxic to the cell are described as potentially lethal lesions (PLLs), which are induced in a domain containing a DNA amount of *g* (kg) in proportional to energy deposition for each domain *z* in Gy (called specific energy). It is assumed that PLLs can transform into lethal lesions (LLs) or be repaired at constant rates as below:

1. A first-order process by which a PLL may transform into an LL at a constant rate of *a* in h^−1^;
2. A second-order process by which two PLLs may interact and transform into an LL at a constant rate of *b*_d_ in h^−1^;
3. A first-order process by which a PLL may be repaired at a constant rate of *c* in h^−1^.

**Figure 1:**
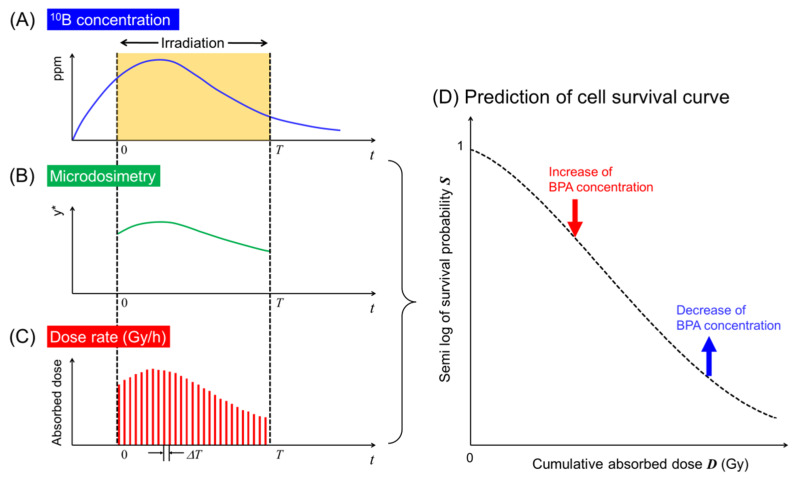
Schematic representation of the timeline of ^10^B concentrations incorporated into the IMK model: (**A**) timeline of the concentration of ^10^B in ppm; (**B**) change of microdosimetric quantity; (**C**) change of dose rate; (**D**) dose-response curve considering changes of microdosimetry and dose rate depending on ^10^B concentration during irradiation. During the course of the irradiation, the concentration of BPA labeled with ^10^B, the microdosimetric quantity and the absorbed dose-rate can be determined by the PHITS calculation. Using the changes in both y* and the absorbed dose per sub-interval of dose-delivery time Δ*T* (i.e., [0, Δ*T*], [Δ*T*, 2Δ*T*], …, [(*N* − 1)Δ*T*, *N*Δ*T*]), the cell survival curve can be described by the IMK model.

Given the energy continuously deposited to the domains during the dose-delivery time *T* in h, we must consider the specific energy (*z*_1_, *z*_2_, …, *z*_N_) and amount of DNA (*g*_1_, *g*_2_, …, *g*_N_) at each sub-section of the dose-delivery time ([0, Δ*T*), [Δ*T*, 2Δ*T*), …, [(*N* − 1)Δ*T*, *NΔT*)) [6,31,33] as shown in Figure 1. Note that the relation T=NΔT can be obtained, where *N* is the number of sub-sections in dose-delivery time *T* in h. By solving the rate equations for PLLs and LLs reported previously [33], the number of LLs per domain *w*_d_, which may lead to cell-killing, can be obtained as follows:

(3) $$\begin{array}{ll} w_{d}= & \sum_{n=1}^{N}\left(A_{n}g_{n}z_{n}\right)+\sum_{n=1}^{N}\left(B_{n}g_{n}{}^{2}z_{n}{}^{2}\right)\\ & +2\sum_{n=1}^{N-1}\sum_{m=n+1}^{N}\left[B_{nm}g_{n}g_{m}z_{n}z_{m}e^{-(m-{n)(a+c}_{n})\Delta T}\right], \end{array}$$

where *A_n_* = *ak*_d_/*c_n_*, *B_n_* = *b*_d_*k*_d_^2^/2*c_n_*, *B_nm_* = 2 *B_n_c_n_*/(*c_n_*+*c_m_*), and *k*_d_ is the PLL induction yield per DNA amount *g* in kg and per specific energy *z* in Gy.

Considering the mean number of LLs per domain <*w*_d_>, the average number of LLs per nucleus <*w*>_T_ can be expressed using the mean dose per nucleus <*z_n_*> = <*D_n_*> and the mean amount of DNA per nucleus <*G_n_*> at a period of dose-delivery time of *t* = (*n−*1)Δ*T* as follows:

(4) $$\begin{array}{lll} {\langle w\rangle }_{T} & = & p\langle w_{d}\rangle \\ & = & \sum_{n=1}^{N}\left(A_{n}p\int_{0}^{\infty }g_{n}{\;f}_{g}{(g}_{n}{)dg}_{n}\int_{0}^{\infty }z_{n}\;f_{z}{(z}_{n}{)dz}_{n}\right)\\ & & +\sum_{n=1}^{N}\left(B_{n}p\int_{0}^{\infty }g_{n}{}^{2}{\;f}_{g}{(g}_{n}{)dg}_{n}\int_{0}^{\infty }z_{n}{}^{2}\;f_{z}{(z}_{n}{)dz}_{n}\right)\\ & & +2\sum_{n=1}^{N-1}\sum_{m=n+1}^{N}[B_{nm}p\int_{0}^{\infty }g_{n}{\;f}_{g}{(g}_{n}{)dg}_{n}\int_{0}^{\infty }z_{n}\;f_{z}{(z}_{n}{)dz}_{n}\\ & & \;\;\;\;\;\;\;\;\;\;\;\;\;\;\;\;\times \int_{0}^{\infty }g_{m}{\;f}_{g}{(g}_{m}{)dg}_{m}\int_{0}^{\infty }z_{m}\;f_{z}{(z}_{m}{)dz}_{m}e^{-(m-{n)(a+c}_{n})\Delta T}], \end{array}$$

(5) $${\langle w\rangle }_{T}=\sum_{n=1}^{N}\left[\left(A_{n}\langle G_{n}\rangle +\gamma_{n}\frac{B_{n}}{p}\langle G_{n}{}^{2}\rangle \right)D_{n}+\frac{B_{n}}{p}\langle G_{n}{}^{2}\rangle D_{n}{}^{2}\right]\;+2\sum_{n=1}^{N-1}\sum_{m=n+1}^{N}\left[\frac{B_{nm}}{p}\langle G_{n}\rangle \langle G_{m}\rangle e^{-(m-{n)(a+c}_{n})\Delta T}\right]D_{n}D_{m},$$

where *p* is the number of domains packaged in the cell nucleus, and

(6) $$D_{n}=\langle z_{n}\rangle =\int_{0}^{\infty }z_{n}\;f_{z}{(z}_{n}{)dz}_{n},$$

(7) $$D_{n}{}^{2}+\gamma_{n}D_{n}=\langle z_{n}\rangle {}^{2}+\frac{y*n}{{\rho \pi r}_{d}^{\;2}}\langle z_{n}\rangle =\int_{0}^{\infty }z_{n}{}^{2}\;f_{z}{(z}_{n}{)dz}_{n},$$

(8) $$\langle G_{n}\rangle =p\int_{0}^{\infty }g_{n}{\;f}_{g}{(g}_{n}{)dg}_{n},$$

(9) $$\langle G_{n}{}^{2}\rangle =p^{2}\int_{0}^{\infty }g_{n}{}^{2}{\;f}_{g}{(g}_{n}{)dg}_{n},$$

(10) $$\langle G_{n}\rangle \langle G_{m}\rangle =p^{2}\int_{0}^{\infty }g_{n}{\;f}_{g}{(g}_{n}{)dg}_{n}.$$

It can be assumed that the cell-cycle dependent parameters (*G* and *c*) for melanoma cells do not change rapidly because of the slow cell-cycle progression (i.e., a doubling time of about 1 day). Thus, we obtain the relations *G_n_* = *G =* constant, *c_n_* = *c =* constant, *A_n_* = *A =* constant, and *B_n_* = *B_n_* = *B =* constant. We can therefore re-define *α*_0_ = *A*<*G*>, *β*_0_ = *B*<*G*^2^>/*p* and $\dot{D}$*_n_∆T = D_n_* for simplicity. Assuming that the number of LLs per nucleus follows a Poisson distribution and that cells have clonogenic ability when <*w*>_T_ = 0, the cell survival probability, *S*, can be expressed as follows:

(11) $$\begin{array}{ll} {\langle w\rangle }_{T}= & \sum_{n=1}^{N}\left[\left(\alpha_{0}+\gamma_{n}*\beta_{0}\right){\dot{D}}_{n}\Delta T+\beta_{0}{({\dot{D}}_{n}\Delta T)}^{2}\right]\\ & \;\;\;\;\;\;+2\sum_{n=1}^{N-1}\sum_{m=n+1}^{N}\left[\beta_{0}e^{-(m-{n)(a+c}_{n})\Delta T}\right]{\dot{D}}_{n}{\dot{D}}_{m}\Delta T^{2}\\ & =-\ln\;S. \end{array}$$

It should be noted that Equation (11) considers the changes in the absorbed dose rate ${\dot{D}}_{n}$ and the microdosimetric quantity $\gamma_{n}*$ depending on the ^10^B concentrations (e.g., 10 or 30 ppm) in the tumor cells during the BNCT irradiations, as shown in Figure 1.

#### 2.2.2. Integrated Microdosimetric-Kinetic Model for a Constant Dose-Rate

From the obtained formula for cell survival probability in the modified IMK model (Equation (11)), we can deduce a simple formula for calculating cell-survival probability after exposure at a constant absorbed dose-rate (${\dot{D}}_{n}$ = $\dot{D}$ = constant) and without the change in microdosimetric quantities ($\gamma_{n}*$ = γ* *=* constant) during irradiation. Based on our previous reports [33], taking the limit *N* to infinity (*∆T*→0), cell-survival probability in the IMK model can be approximately expressed by

(12) $$\begin{array}{l} -\ln\;S=\left(\alpha_{0}+\gamma *\beta_{0}\right)\dot{D}T+\frac{2\beta_{0}}{{\left(a+c\right)}^{2}T^{2}}\left[{(a+c)T+e}^{-(a+c)T}-1\right]{\left(\dot{D}T\right)}^{2}\\ =\alpha D+\beta D^{2} \end{array}$$

where

(13) $$\alpha =\alpha_{0}+\gamma \beta_{0},$$

(14) $$\beta =\frac{2\beta_{0}}{{\left(a+c\right)}^{2}T^{2}}\left[{(a+c)T+e}^{-(a+c)T}-1\right],$$

(15) $$D=\dot{D}T.$$

It is notable that Equation (12) is linked to the formula including the Lea–Catchesides time factor [42] for describing dose-rate effects (e.g., sparing effects for low-dose-rate irradiation). We used Equation (12) for determining the model parameters in the IMK model for melanoma cells and compared the calculated dose–response curves to the experimental data for ^60^Co γ-rays, thermal neutrons only, and BNCT irradiations.

### 2.3. Determination of Model Parameters for Human Melanoma

We determined sets of model parameters for three types of human melanoma cells—the HX43, M8 and Mel-J cell lines—for ^60^Co γ-rays (*y_D_* = 2.26 keV/μm [34] ≅ y* [21])), using a simulation technique with Markov chain Monte Carlo (MCMC) [31,43,44]. In the MCMC sampling simulation, the IMK model (Equation (12)) consists of the set of parameters *θ*(*α*_0_, *β_0_*, (*a + c*) 1/σ)), where *σ* is the standard deviation of −ln *S.* In accordance with the MCMC algorithms reported previously [31], we sampled the set of parameters under the assumption that the uncertainty for −ln *S* follows a normal distribution. The prior distributions of *α*_0_ and *β_0_* were set to be uniform, while that of (*a + c*) was obtained from a significant number of dose rate data on human melanoma HX34 cells (as shown in Figure 3A in Results and Discussion). We first determined the model parameters of the HX34 cell line following the likelihood function *P*(*d_i_*|*θ*) and the transition probability *α*_P_, as follows:

(16) $$\;P(d\;|\theta )=\overset{N}{\underset{i=1}{\prod }}\left[P(d_{i}\;|\theta )\right]=\overset{N}{\underset{i=1}{\prod }}\left\{\frac{1}{\sqrt{2\pi \sigma }}\exp\left[-\frac{{\left(-{\ln\;S}_{\mathrm{expi}\;}{+\ln\;S}_{\mathrm{modi}}\right)}^{2}}{{2\sigma }^{2}}\right]\right\}$$

(17) $$\alpha_{P}=\frac{{P(\theta \;}^{candidate}\left|\;d\right)}{P(\theta {\;}^{\left(t\right)}\left|\;d\right)}$$

where *d_i_* (*i* = 1~*N*) is the set of experimental data represented as the vector (*D_i_*, −ln *S*_exp*i*_), *S*_exp_ is the measured cell survival probability, *S*_mod_ is the value calculated by the IMK model, and *P*(*θ*|*d*) and *P*(*θ^candidate^*|*d*) are the posterior probabilities for the candidate (*t* + 1)th and the previous (*t*)th conditions, respectively. Comparing the random number (0–1) to the acceptance ratio *α*_P_, we sampled 10^4^ sets of model parameters for each cell line. It should be noted that we also set the burn-in to be 10^3^ to exclude the dependency of initial parameters on posterior parameters. Using the posterior value of (*a + c*) for the HX34 cell line [45], which we determined as 8.857 ± 2.175 (h^−1^) (see Table 1), we also determined the set of model parameters for the M8 and Mel-J cell lines based on the experimental survival data after irradiation with ^60^Co γ-rays. Note that the method of updating the model parameters is based on the Bayesian theory.

### 2.4. Mean Inactivation Dose and Relative Biological Effectiveness

To compare this work to the corresponding experimental data including recommended data, we calculated the relative biological effectiveness (RBE) at the endpoint of mean inactivation dose (MID). In the MID concept recommended by the ICRU Report 30 [46], the dose–response curve for cell survival is treated as a probability distribution of cell-killing with the absorbed dose. The MID, represented as $\bar{D}$, means the mean dose necessary to inactivate cells, which is given by

(18) $$\bar{D}=\int_{0}^{\infty }S(D)dD,$$

where *S*(*D*) is the survival probability. The $\bar{D}$ values for various dose rates were calculated based on Equations (11) and (12). Taking the $\bar{D}$ ratio of photon beams and any radiation type (e.g., thermal neutron or BNCT), we calculated the RBE value as follows:

(19) $$\mathrm{RBE}=\frac{{\bar{D}}_{\mathrm{photon}}}{{\bar{D}}^{*}},$$

where ${\bar{D}}_{\mathrm{photon}}$ is the MID for photon beams, used as a reference radiation at an extremely high dose-rate (i.e., 10 Gy/min), and ${\bar{D}}^{*}$ is the MID for any type of radiation. Using this RBE value, we evaluated the impact of the dose-rate on the curative effects (cell-killing) after thermal neutron irradiation and BNCT irradiation.

## 3. Results and Discussion

### 3.1. Comparison between In Vitro Experimental Data and Corresponding Model Predictions

We first test whether the present model can reproduce in vitro experimental survival data for primary cutaneous malignant melanoma and metastatic melanoma. The microdosimetric quantities (dose-mean lineal energy *y_D_* in keV/μm, the saturation-correlated value considering the over-kill effects for high-LET radiation y* in keV/μm) for ^60^Co γ-rays, thermal neutron irradiation and BNCT in the presence of 10 ppm BPA were calculated using the Monte Carlo simulation code of the Particle and Heavy Ion Transport code System (PHITS) version 3.11 [28]. Using these values, we calculated the cell-survival probability for ^60^Co γ-rays, thermal neutron irradiation, and BNCT and compared them to experimental data for the melanoma cell lines M8 and Mel-J [39].

**Figure 2:**
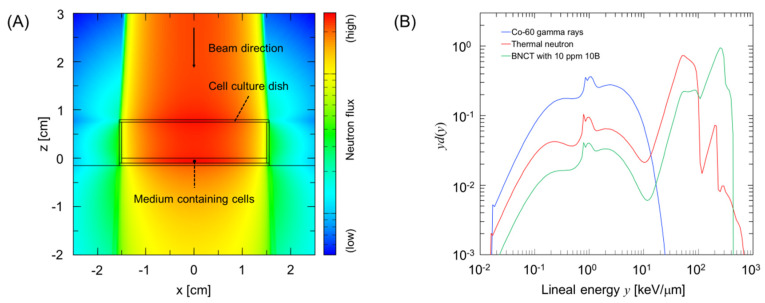
Calculation of the microdosimetric quantities of *y_D_* and y* for thermal neutrons and boron neutron capture therapy (BNCT) irradiations. (**A**) shows the simulation geometry illustrated by the Particle and Heavy Ion Transport code System (PHITS) code [28], and (**B**) shows examples of relationships between lineal energy *y* and probability density of dose *d*(*y*) in a domain with a diameter of 1 μm for calculating *y_D_* and y*. These values were used to calculate cell-survival probability using the integrated microdosimetric-kinetic (IMK) model.

Assuming that a cell culture (petri) dish with 30 mm diameter was exposed to radiation, we evaluated the microdosimetric quantities, as shown in Figure 2. Figure 2A shows the simulation geometry considered in the PHITS calculation, and Figure 2B shows the calculated probability density of dose for lineal energy *y* in keV/μm.

**Figure 3:**
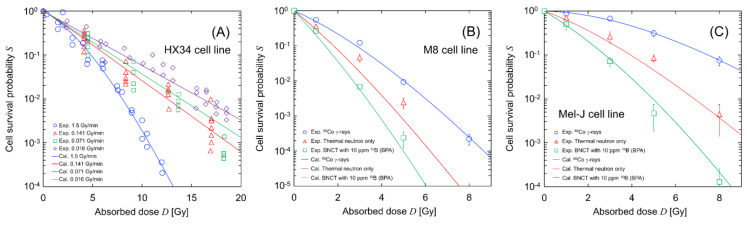
Calculated cell survival curve after irradiations: (**A**) dose-rate effects under ^60^Co γ-ray irradiation; (**B**) dose–response curves for the M8 cell line; and (**C**) dose–response curve for the Mel-J cell line. These experimental data were obtained from the literature [39,45], while the predicted curves were described using the model parameters listed in Table 1 and the IMK model. Dose-rates and microdosimetric quantities for ^60^Co γ-rays, thermal neutron only, and BNCT with 10 ppm BPA are 1.25 Gy/min and 2.26 keV/μm, 1.0 Gy/min and 41.36 keV/μm, and 3.75 Gy/min and 68.50 keV/μm, respectively. Note that the dose rate for BNCT was calculated from the dose rate for the neutron-only irradiation and the PHITS calculation.

Figure 3 compares the calculated cell-survival rates (solid lines) with the experimental data (symbols) [37,38]; (A) represents the dose-rate dependency (which is the result from SLDR (repair of potentially lethal lesions leading to cell death with certain probability [33,47,48,49,50]) during ^60^Co γ-ray irradiation) of HX34 melanoma, (B) shows the dose–response curves for the primary cutaneous malignant M8 melanoma, and (C) shows the curves for the Mel-J cells from a metastatic melanoma lesion of the lung. It should be noted that the curves in Figure 3A and the blue lines in Figure 3B,C are fitting curves of the IMK model to the experimental data. The dose rate for BNCT with 10 ppm BPA was estimated from the PHITS calculation. The model parameters and their uncertainties for each cell line were obtained during the fitting processes by Markov chain Monte Carlo (MCMC) simulations [31], which are summarized in Table 1.

The results presented in Figure 3A show that the SLDR rate of melanoma was extremely high, at 8.86 ± 2.18 (h^−1^). They also show that the y* values for thermal neutron irradiation and BNCT (i.e., 41.36 keV/μm for neutron-only irradiation and 68.50 keV/μm for BNCT) are important parameters for reproducing the experimental survival probability for BNCT irradiations.

To obtain the y* value for BNCT, we assumed that ^10^B is uniformly distributed in cells because BPA can enter cells through amino acid transporters on the cell membrane surface. The heterogeneous nature of ^10^B concentration in tumors should be considered for evaluating the killing of cancer cells [26]. In addition, a subtle difference in the thermal neutron spectrum between facilities and the gamma contamination in neutron facilities (which is not considered in this simulation) can also potentially affect survival curves. However, this simple and approximate approach is still able to reproduce the experimental dose responses for thermal neutron irradiation with or without BPA labelled with ^10^B. This comparison of the model and the experimental data [39] proves that the MK model coupled with y* can effectively predict the cell-survival probability for BNCT. The most recent technique of proton boron capture therapy, in which the p + ^11^B → 3α reaction enhances the radiosensitivity of tumors [51,52], has been biologically reported; thus, further model studies for capture therapy with boron are necessary in the future.

### 3.2. Dependence of ^10^B Concentration in Tumor Cells on Biological Effects

The y* value for neutron irradiations with any BPA concentration can be obtained from a Monte Carlo simulation using the PHITS code. In addition to the comparison in Figure 3, we estimated the radiosensitivity for various BPA concentrations. The relationship between the BPA concentration and the relative biological effectiveness (RBE) was calculated using this model for each cell line and compared to the RBE values reported in the literature [25,26,53,54,55].

**Figure 4:**
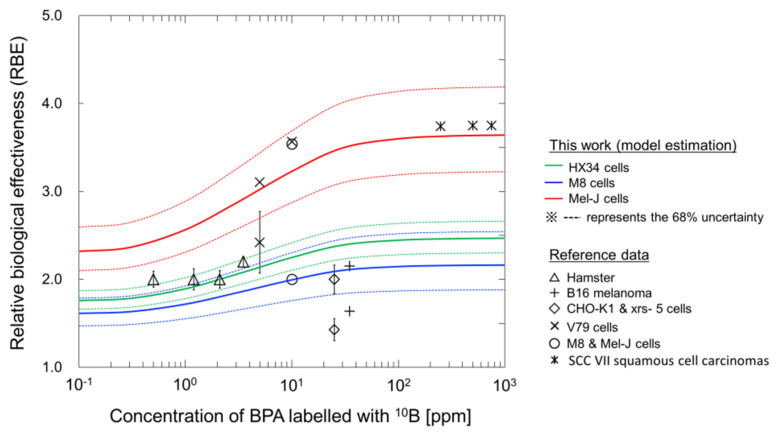
Relationship between ^10^B-boronphenylalanine (BPA) concentration and relative biological effectiveness (RBE); solid lines are the estimations from the model, and the symbols are the recommended RBE values reported in the literature [25,26,53,54,55]. To calculate the RBE values, the y* values for neutron irradiations with any BPA concentration were obtained from the PHITS calculation [28]. The dotted lines represent the 68% uncertainties calculated from the set of model parameters obtained by the MCMC simulation.

Figure 4 shows the dependency of BPA concentration in the tumor cells on the RBE value for the cell lines HX34 (green line), M8 (blue line) and Mel-J (red line). The RBE value for each cell line was calculated from the ratio of the mean inactivation dose (MID) for photon beams to that for BNCT. The MID, which is the mean dose necessary to inactivate cells, is recommended in the ICRU Report 30 [46]. As shown in Figure 4, the RBE increases as the concentration of BPA increases for all cell types, while the minimum and maximum RBE values largely depend on the type of cell line. Using this comparison, the present model enables us to obtain the recommended RBE value for BNCT irradiations [25,26,53,54,55].

### 3.3. Dose-Rate Effects under BNCT Irradiations

The present model enables us to evaluate cell recovery during irradiation (dose-rate effects). Assuming that the dose rate during BNCT irradiation is constant in vitro, we estimated the RBE at the MID as a function of the dose rate. The estimated RBE values were then compared to many available experimental dose rate data for the various cell lines [25,33,39,45,56,57,58]. Experimental data on BNCT irradiation in the presence of around 10 ppm (5–20 ppm) [25,39,57] were used for this comparison.

**Figure 5:**
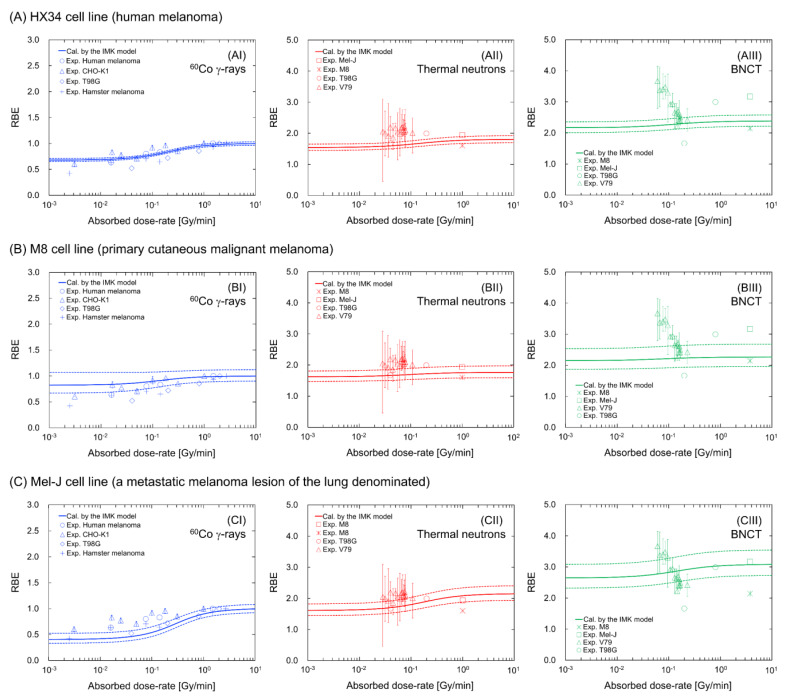
Estimation of dose-rate effects on RBE values for ^60^Co g-rays, neutron only and BNCT in the presence of 10 ppm BPA labeled with ^10^B for the (**A**) HX34 cell line, (**B**) M8 cell line and (**C**) Mel-J cell line. For the BNCT irradiation, we assumed that ^10^B concentrations in cells are constant. The solid and dotted lines represent the mean value and 68% confidence intervals, respectively, which are calculated by MCMC sampling. The symbols represent the experimental data obtained from the literature [25,33,39,45,56,57,58].

Figure 5 shows the dose-rate dependency on the RBE value for (A) the HX43 cell line, (B) the M8 cell line and (C) the Mel-J cell line. The solid and dotted lines represent the mean RBE value and 68% confidence intervals (CI), respectively, which were calculated by the set of model parameters obtained by the MCMC simulation. The results for the ^60^Co γ-rays in the left panels of Figure 5 show that the RBE value decreases as the dose rate decreases (e.g., 0.824 (68% CI: 0.668–1.07) for the M8 cell line and 0.406 (68% CI: 0.332–0.523) for the Mel-J cell line, at the lowest dose rate of 10^−3^ Gy/min). Meanwhile, the decrease in the RBE values under neutron-only irradiation (central panels) and BNCT irradiation (right panels) is less than that under ^60^Co γ-ray irradiation. The tendencies for ^60^Co γ-rays and thermal neutron only are in good agreement with the corresponding experimental data for the various cell lines; however, there are large discrepancies in the case of BNCT irradiation at dose-rates lower than approximately 0.1 Gy/min (as shown in the right panels in Figure 5). This may be caused by inverse-dose-rate effects (IDREs) [59,60,61]. It has been suspected that IDREs can occur due to changes in radiosensitivity resulting from cell-cycle dynamics [33,62,63], or cumulative low-dose hyper-radiosensitivity induced by a failure to arrest in G_2_ [64,65,66] during long-term (protracted) irradiation. Considering the significant experimental uncertainty in the BNCT data and the uncertain mechanisms of IDREs, further in vitro investigations to clarify the involvement of IDREs in enhanced radiosensitivity at low-dose-rates are required.

The model estimations shown in Figure 5 suggest a reduced impact of cell recovery during high-LET BNCT irradiations compared to that with low-LET ^60^Co γ-rays irradiation. The small impact of dose-rate effects is attributed to the methodology of the MK model (i.e., the increase in the y* (*y_D_*) value with a constant *β*_0_ value) [18,19]. This assumption is reasonable for reproducing the cell-survival probability for various LET radiations based on numerous studies coupled with the MK model [18,19,21,22,23,24,25,26,27]. By contrast, a recent model approach uses a variable *β*_0_ value dependent on the LET value [67]. It is suspected that the coefficient of *β*_0_ is closely connected with the LET-dependent DNA lesion (e.g., DNA double-strand break) yield [68,69,70]. To precisely interpret the biological responses, further model development with in vitro experiments is necessary in future studies.

### 3.4. Estimation of Dose–Response Curve Considering the Dynamics of BPA Concentrations

The reasonable agreement of the present model with the experimental data [25,33,39,45,56,57,58] for irradiation cases at a constant dose-rate (Figure 3 and Figure 5) demonstrates that the model enables us to evaluate the impact of both cell recovery and ^10^B concentrations on the dose–response curve for cell-survival probability. As a final step, we tried to describe the nature of the dose–response curve considering ^10^B concentrations dynamics using an example of ^10^B concentrations dynamics after injection [71]. It should be noted that we focused on both changes in microdosimetric quantity and cell recovery resulting from SLDR, excluding the IDREs.

**Figure 6:**
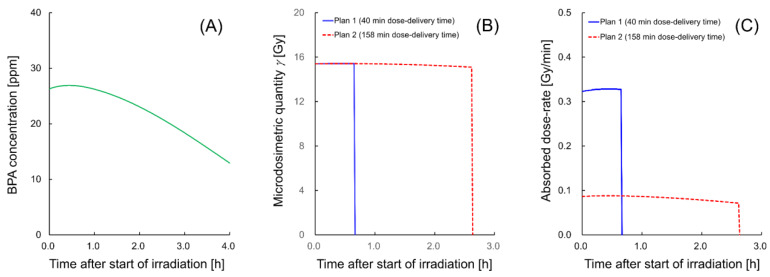
Timelines of the (**A**) ^10^B concentration, (**B**) microdosimetric quantity and (**C**) absorbed dose rate after the start of irradiation. These example irradiation conditions exemplify the impact of ^10^B concentration dynamics during irradiation (2 h after BPA injection [71]) on malignant melanoma. With reference to the literature, i.e., an experimental protocol [71] and a clinical report [72], we set out two irradiation regimens, one with a 40 min dose-delivery time and the other with a 158 min dose-delivery time, to deliver 13.86 Gy as a total absorbed dose (which is calculated from the RBE-Gy reported in the literature [71]).

Figure 6 shows the BNCT irradiation conditions, where (A) is the timeline of BPA concentrations after the start of irradiation, which was obtained from the measured data on the BPA concentrations after injection reported in the literature [71], (B) is the change in microdosimetric quantity during irradiation and (C) is the change in the absorbed dose rate during irradiation. It should be noted that the green solid line representing BPA concentrations is described from the experimental measurements [71] and spline interpolation. The microdosimetric quantity and dose-rate shown in Figure 6B,C were calculated by the PHITS code. With reference to the literature, i.e., an experimental protocol [71] and a clinical report [72], we prepared two examples of irradiation regimens, one with a 40 min dose-delivery time and the other with a 158 min dose-delivery time, to deliver 13.86 Gy (which is a physical dose calculated from the literature [71]) to melanoma in single-dose irradiation.

**Figure 7:**
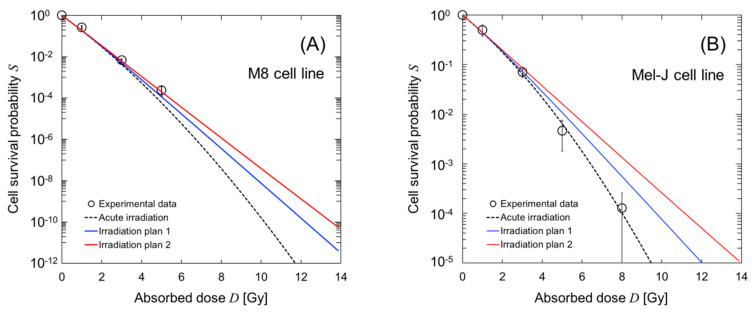
Estimation of the dose–response curve for cell survival considering the ^10^B concentration dynamics during BNCT irradiation. To estimate the curves, we used the model parameters for the M8 and Mel-J cell lines (Table 1) and the irradiation regimens described in Figure 6. (**A**,**B**) shows the curves for the M8 and Mel-J cell lines, respectively. The symbols are the experimental data measured using high-dose-rate neutrons at 1.0 Gy/min [39].

For the irradiation courses, we calculated the dose–response curves for cell survival in the M8 (malignant tumor) and Mel-J (metastatic melanoma) cell lines. Figure 7 shows the estimated dose–response curves for cell survival; (A) is the curve for the M8 cell line and (B) is that for the Mel-J cell line. As shown in Figure 7, the dose–response curves for the short dose-delivery time of 40 min (Plan 1, represented by a blue solid line) shows a high-dose radio-resistance greater than that shown by the curve after acute irradiation (which was calculated using the averaged concentration of BPA during irradiation). The curve for the long dose-delivery time of 158 min (Plan 2) exhibits significant cell recovery.

These model estimations, even for the case of BNCT with a reduced impact of cell recovery during irradiation, suggest that the dose-rate effects resulting from the ^10^B concentration dynamics cannot be ignored when treating melanoma with BNCT. These results from the present model would contribute to predicting cell recovery in a more realistic dose-delivery scheme in BNCT.

## 4. Conclusions

The tumor-cell-selective killing kinetics are recognized as an important issue when discussing the effectiveness of boron neutron capture therapy (BNCT) irradiation. In this paper, we present a cell-killing model, the *integrated microdosimetric-kinetic* (*IMK*) *model*, which considers the time-dependencies of ^10^B concentrations in cancer cells and DNA lesion kinetics (the sub-lethal damage repair process) during reactor- and accelerator-based BNCT irradiations. The development of the model and its estimation coupled with the Particle and Heavy Ion Transport code System show that the microdosimetric quantities of *y_D_* and y* in keV/μm are important for reproducing dose–response curves of cell survival for BNCT irradiation. The model exhibits a reduced impact of cell recovery during BNCT irradiation, which may be of advantage compared to photon irradiations. In the accelerator-based BNCT era, more patients can easily access BNCT at medical institutes, and the unique property of tumor-cell-selective irradiation with heavy particles will improve the clinical outcomes of cancer treatment [10]. Our model can contribute to an understanding of the importance of ^10^B concentration dynamics during irradiation and enable us to predict the radiosensitivity in more realistic treatment planning that takes into account not only drug delivery but also dose delivery in BNCT. Because of the limited amount of available experimental data, further dose rate experiments in vitro are essential for the more precise estimation of cellular responses in future studies of BNCT.

## Figures and Tables

**Table 1:** Model parameters determined by Markov chain Monte Carlo (MCMC) simulation of human melanoma.

Cell Line Type	Parameters	Values	Unit	How to Obtain the Parameter’s Values
HX34 cell line	*α*_0_	0.263 ± 0.016	Gy^−1^	MCMC with ref. [45] (^60^Co γ-ray data)
	*β*_0_	0.047 ± 0.005	Gy^−2^	MCMC with ref. [45] (^60^Co γ-ray data)
	(*a + c*)	8.857 ± 2.175 *	h^−1^	MCMC with ref. [45] (^60^Co γ-ray data)
M8 cell line (primary melanoma)	*α*_0_	0.612 ± 0.130	Gy^−1^	MCMC with ref. [39] (^60^Co γ-ray data)
	*β*_0_	0.066 ± 0.020	Gy^−2^	MCMC with ref. [39] (^60^Co γ-ray data)
	(*a + c*)	8.769 ± 2.128	h^−1^	Update with ref. [39] and the (*a + c*) value *
Mel-J cell line (metastatic melanoma)	*α*_0_	0.002 ± 0.047	Gy^−1^	MCMC with ref. [39] (^60^Co γ-ray data)
	*β*_0_	0.050 ± 0.009	Gy^−2^	MCMC with ref. [39] (^60^Co γ-ray data)
	(*a + c*)	8.916 ± 2.126	h^−1^	Update with ref. [39] and the (*a + c*) value *

*: The (*a + c*) value used for update is that determined from the MCMC and experimental data of HX34 cells.
